# EPIDEMIOLOGICAL INVISIBILITY CHARACTERIZES BRAZILIAN NATIVE INDIANS’ HEALTH

**DOI:** 10.1590/1984-0462/;2018;36;2;00018

**Published:** 2018

**Authors:** Douglas A. Rodrigues

**Affiliations:** a Departamento de Medicina Preventiva, Universidade Federal de São Paulo, São Paulo, SP, Brasil.

Indigenous peoples in Brazil are minorities living in situation of exclusion, marginalization, and discrimination, which increase their vulnerability to health problems. Upon the arrival of the Portuguese and the Spanish in South America, the Guaranis were a group of people of the same origin, speakers of the same language with dialectical variations from group to group and keeping a way of being that preserved the memory of their ancient traditions. They practiced a very productive agriculture, including corn, cassava, potatoes, peanuts, grains, pumpkins, pineapples, and other crops. Over the centuries, they had their lands plundered and currently survive in a region widely exploited by modern agroindustry, with no room to keep reproducing cultural practices in their communities. They are coerced into living in overpopulated reserves, without basic sanitation and in substandard housing. The lack of lands and the exhaustion of the existing fields have led the Guaranis to seek livelihood in wage labor, whether with underemployment in urban areas or at sugar and ethanol farms and factories. This directly reflects in morbidity and mortality indicators among the Guaranis, being two to three times higher than data registered nationally. Hunger, malnutrition, occupational hazards, poor access to health services, and social violence are some of the main health-condition determinants.

Seen in these terms, the publication of the article “Acute lower respiratory infection in Guarani children - Brazil”,^1^ in this issue of *Revista Paulista de Pediatria* is extremely timely*.* This study makes a valuable contribution to reducing the epidemiological invisibility that has characterized the health of indigenous peoples in Brazil, on account of the difficulty in obtaining reliable information from official health databases. It also points out that, although they have been given wider access to health services after the creation of the Subsystem for Indigenous Health Care of the public health system (SUS), formed by a network of Special Indigenous Health Districts aimed at providing primary health care benefits, these facilities have been insufficient to ensure good health conditions for users. Therefore, intersectoral policies, especially land-related, capable of minimizing this situation are needed. Providing them with knowledge about their health conditions and communication with health service centers is extremely important for the evaluation of public policies aimed at this audience and to support the necessary interventions intended for promoting health equity between indigenous and non-indigenous people in our country.
